# Acacetin inhibits invasion, migration and TGF-β1-induced EMT of gastric cancer cells through the PI3K/Akt/Snail pathway

**DOI:** 10.1186/s12906-021-03494-w

**Published:** 2022-01-09

**Authors:** Guangtao Zhang, Zhaoyan Li, Jiahuan Dong, Weili Zhou, Zhanxia Zhang, Zujun Que, Xiaohong Zhu, Yan Xu, Nida Cao, Aiguang Zhao

**Affiliations:** 1 Department of Oncology, Longhua Hospital, Shanghai University of Traditional Chinese Medicine, Shanghai, 200032 China; 2 Department of Oncology, Yueyang Hospital, Shanghai University of Traditional Chinese Medicine, Shanghai, China; 3 Institute of Traditional Chinese Medicine Oncology, Longhua Hospital, Shanghai University of Traditional Chinese Medicine, Shanghai, China; 4 School of Oncology, Shanghai University of Traditional Chinese Medicine, Shanghai, China

**Keywords:** Epithelial-to-mesenchymal transition (EMT), Acacetin, Gastric cancer (GC), TGF-β1, Metastasis

## Abstract

**Background:**

Epithelial-to-mesenchymal transition (EMT) is a pivotal cellular phenomenon involved in tumour metastasis and progression. In gastric cancer (GC), EMT is the main reason for recurrence and metastasis in postoperative patients. Acacetin exhibits various biological activities. However, the inhibitory effect of acacetin on EMT in GC is still unknown. Herein, we explored the possible mechanism of acacetin on EMT in GC *in vitro* and *in vivo*.

**Methods:**

*In vitro,* MKN45 and MGC803 cells were treated with acacetin, after which cell viability was detected by CCK-8 assays, cell migration and invasion were detected by using Transwell and wound healing assays, and protein expression was analysed by western blots and immunofluorescence staining. *In vivo*, a peritoneal metastasis model of MKN45 GC cells was used to investigate the effects of acacetin.

**Results:**

Acacetin inhibited the proliferation, invasion and migration of MKN45 and MGC803 human GC cells by regulating the expression of EMT-related proteins. In TGF-β1-induced EMT models, acacetin reversed the morphological changes from epithelial to mesenchymal cells, and invasion and migration were limited by regulating EMT. In addition, acacetin suppressed the activation of PI3K/Akt signalling and decreased the phosphorylation levels of TGF-β1-treated GC cells. The *in vivo* experiments demonstrated that acacetin delayed the development of peritoneal metastasis of GC in nude mice. Liver metastasis was restricted by altering the expression of EMT-related proteins.

**Conclusion:**

Our study showed that the invasion, metastasis and TGF-β1-induced EMT of GC are inhibited by acacetin, and the mechanism may involve the suppression of the PI3K/Akt/Snail signalling pathway. Therefore, acacetin is a potential therapeutic reagent for the treatment of GC patients with recurrence and metastasis.

**Supplementary Information:**

The online version contains supplementary material available at 10.1186/s12906-021-03494-w.

## Background

Gastric cancer (GC) has the fifth highest incidence worldwide and the third highest mortality rate [1]. The prognosis of GC is poor because most patients are diagnosed at an advanced stage or because frequent recurrence and distant metastasis occur after surgical resection [2]. Peritoneal metastasis is the most common pattern of GC after standard radical resection, and the median survival time of patients is very short [3]. Moreover, the liver is the most common site of haematogenous metastasis of GC, and up to 37% of GC patients developed hepatic metastases after radical gastrectomy [4]. After metastasis in GC, fewer effective treatments are available.

EMT is a reversible cellular process that transiently places epithelial cells into quasi-mesenchymal cell states [5]. This process facilitates the motility of adherent epithelial cells and endows cells with migratory properties via reduced adhesion and added invasive capacity; most importantly, the mesenchymal state is associated with the capacity of cells to migrate to distant organs during metastasis [6, 7]. Indeed, tumours often express EMT markers or lose epithelial markers prior to the invasive process and extravasation of circulating tumour cells [8]. Many factors, such as TGF-β, epidermal growth factor, fibroblast growth factor, Notch pathways, cytokines, extracellular matrix components or mechanotransduction, can induce EMT [9–13]. In response to these extracellular signals, epithelial cells achieve dramatic changes in motility.

Transforming growth factor-β1 (TGF-β1) is the ligand of the TGF-β receptor complex, and activation of the TGF-β signalling pathway plays a crucial role in many biological programs, such as the genesis, development and metastasis of tumours [14, 15]. TGF-β signalling was also shown to play a central role in EMT [16], where it activates SMADs and collaborates with other signalling cascades, such as ERK, p38 MAPK and PI3K/Akt [17, 18]. The PI3K/Akt pathway can regulate cell proliferation and maintain malignant biological behaviour [19]. Activation of this pathway is a central feature in EMT [20]. Therefore, PI3K/Akt pathway-mediated EMT has led to continuing concern, as it acts as a possible target for the prevention and treatment of metastatic tumours.

Acacetin (5,7-dihydroxy-4′-methoxyflavone) is a flavone that is widely present in various plants, such as black locust, *Pseudostellaria heterophylla*, *Chrysanthemum indicum*, and patchouli. This molecule exhibits various biological activities, including anticancer, antimicrobial, antioxidant, anti-inflammatory, antimalarial, antiobesity, and vasorelaxant activities [21–23]. Previous studies have shown that acacetin inhibits invasion and metastasis in various cancer cell lines, including lung cancer and prostate cancer cell lines [24–26]. In breast cancer cells, acacetin was shown to inhibit cell adhesion, focal adhesion formation and migration in a concentration-dependent manner [27]. However, the role of acacetin in EMT in GC is still unclear. In this study, we elucidated the anticancer mechanism of acacetin in GC *in vitro* and *in vivo*.

## Materials and methods

### Reagents

Acacetin (purity 98%) was purchased from Tauto Biotech Co., Ltd. (Shanghai, China). RPMI 1640 medium was obtained from Gibco (Beijing, China). Foetal bovine serum was purchased from Gibco (CA, USA). Cell Counting Kit-8 (CCK-8) reagent was purchased from Dojindo (Dojindo Laboratories, Japan). TGF-β1 was purchased from R&D Systems (Minneapolis, MN, USA). Anti-p-PI3K (Tyr458), anti-p-Akt (Ser473), anti-PI3K and anti-Akt were purchased from Affinity Biosciences. Anti-MMP-9, anti-MMP-2 and anti-Snail were purchased from Cell Signaling Technology (Danvers, MA, USA). Anti-Vimentin, anti-N-cadherin, anti-E-cadherin, anti-GAPDH, and horseradish peroxidase (HRP)-conjugated secondary antibodies were obtained from Proteintech (Wuhan, China).

### Cell lines

The human GC cell lines MKN45 and MGC803 were purchased from the Shanghai Institute of Cell Biology, Chinese Academy of Sciences (Shanghai, China). The cells were cultured in RPMI-1640 medium containing 10% foetal bovine serum and 1% penicillin/streptomycin and incubated at 37 °C with 5% CO_2_.

### Cell viability assay

The viability of the GC cell lines MKN45 and MGC803 was detected by CCK-8 assays. Acacetin was dissolved in dimethylsulfoxide (DMSO) at a concentration less than 3‰. MKN45 and MGC803 cells were uniformly plated in 96-well plates at a density of 3 × 10^3^ cells/well. The cells were treated with different concentrations of acacetin (from 0 to 100 μM). Then, the cytotoxicity of the cells cultured with acacetin was assessed at 24, 48, and 72 h. CCK-8 solution (10 μl/well) was incubated for 1 h, and the absorbance was measured at 450 nm using a microplate reader (BioTek, America).

### Migration and invasion assays

For the migration assay, the cells (2 × 10^4^) were collected and seeded in the upper chamber (8 μm) in 24-well plates (Corning) with 300 μl of serum-free medium containing acacetin (40 μM) or 10 ng/ml TGF-β1. For invasion experiments, diluted (1:3) basement Matrigel was added to each chamber and allowed to polymerize at 37 °C for 30 min. The cells (1 × 10^5^) were seeded in the upper chamber with 300 μl of serum-free medium containing acacetin (40 μM) or 10 ng/ml TGF-β1. FBS medium (20%) was added to the lower chamber of the Transwell. After incubation for 24 h, cells on the top of the chamber were removed with cotton swabs, and the migrated cells were stained with 0.5% crystal violet. The Transwell was photographed under a microscope (Leica Microsystems, Wetzlar, Germany) at 20× magnification. Five fields were randomly selected for quantification. The number of invasive cells was counted according to the cell morphology because the nucleus was clearly visible.

### Wound healing assay

MKN45 and MGC803 cells were cultured in 6-well plates overnight. The cell monolayers were wounded by scratching with 10 μl sterile pipette tips and washed with PBS. Fresh serum-free medium containing TGF-β1 (10 ng/ml) with or without acacetin (40 μM) was added to the plates, and then, the cells were cultured for 24 h at 37 °C. Images of the different stages of wound healing were obtained via microscopy at different time intervals. The wounded areas were quantified by wound width using ImageJ software (National Institutes of Health, Bethesda, MD, USA).

### Immunofluorescence staining

The cells were seeded in glass bottom cell culture dishes (Nest), treated with acacetin (40 μM), fixed in 4% paraformaldehyde at room temperature for 30 min, and then washed three times with PBS. BSA (2%) in PBS was used to block the cells for 1 h, and the cells were incubated with primary antibodies (E-cadherin 1:200; N-cadherin 1:200) overnight at 4 °C, washed three times with PBS, and incubated with FITC-conjugated secondary antibodies (1:200 dilution, Sigma-Aldrich, St. Louis, MO) for 2 h. Nuclear staining was performed with 1 μg/ml 4′,6-diamidino-2-phenylindole (DAPI). Images were examined with a fluorescence microscope.

### Western blot

For western blot analysis, total protein samples were extracted from MKN45 or MGC803 cells, and liver metastatic tumour tissues were collected from nude mice using a protease inhibitor to break up the cells in a homogenizer. An equal amount of protein (30 μg/lane) was loaded on 10% SDS-polyacrylamide gels and then transferred to a pure polyvinylidene difluoride membrane. Some membranes were cut according to the molecular weight of the test proteins. Next, the membrane was blocked in 5% nonfat milk for 1.5 h and then probed with primary antibodies against PI3K, p-PI3K, Akt, p-Akt, Snail, E-cadherin, N-cadherin, Vimentin, MMP2, MMP9, and GAPDH. Then, the membranes were incubated with anti-mouse or anti-rabbit secondary antibodies for 2 h. Finally, immunoreactivity was detected using the Tanon Imaging System (Shanghai, China). The densitometric estimations were quantified using ImageJ software (National Institutes of Health, Bethesda, MD, USA). All of the raw data using western blotting were supplemented in Figs. S1, S2, S3, S4, S5, S6 and S7.

### Animal experiments

The study was carried out in accordance with the Declaration of Helsinki. All experiments were conducted following the Guide for the Care and Use of Laboratory Animals approved by Longhua Hospital, Shanghai University of Traditional Chinese Medicine (LHERAW-2003). Male BALB/c nude mice (21–25 g weight; 4–6 weeks old) were purchased from GemPharmatech Co., Ltd. (Nanjing, China) and maintained in specific pathogen-free (SPF) conditions in accordance with institutional policies. The mice received sterile rodent chow and water ad libitum and were housed in sterile filter-top cages with 12-h light/dark cycles. Human MKN45 cells were collected, resuspended in PBS and injected intraperitoneally into the mice (2 × 10^6^ cells/mouse, *n* = 5). The mice were regularly fed. On the 8th day after modelling, acacetin was administered intraperitoneally. Acacetin was dissolved in DMSO and diluted with cyclodextrin at the time of injection, and the concentration of DMSO was not greater than 5%. Nude mice were divided into three groups: the control group (solvent only), acacetin low-dose group (25 mg/kg) and acacetin high-dose group (50 mg/kg). The frequency of administration was once every two days. During the experiments, the body weight of each mouse was measured 2–3 times every week. The mice were sacrificed approximately 3 weeks later, the abdominal colonized tumours were counted, and the number of metastatic nodules in the livers was observed. The histological features of liver metastatic nodules in different groups of mice were analysed through routine pathological staining.

### Statistical analysis

All data are expressed as the mean ± SEM. Statistical analyses were performed using Student’s t-test for two group comparisons. GraphPad Prism 5.0 was used for statistical analyses. A value *p < 0.05, **p < 0.01 or ***p < 0.001 and # *p* < 0.05, ## *p* < 0.01, or ### *p* < 0.001 was considered statistically significant.

## Results

### Acacetin inhibited the proliferation, invasion and migration of MKN45 and MGC803 cells

**Fig. 1:**
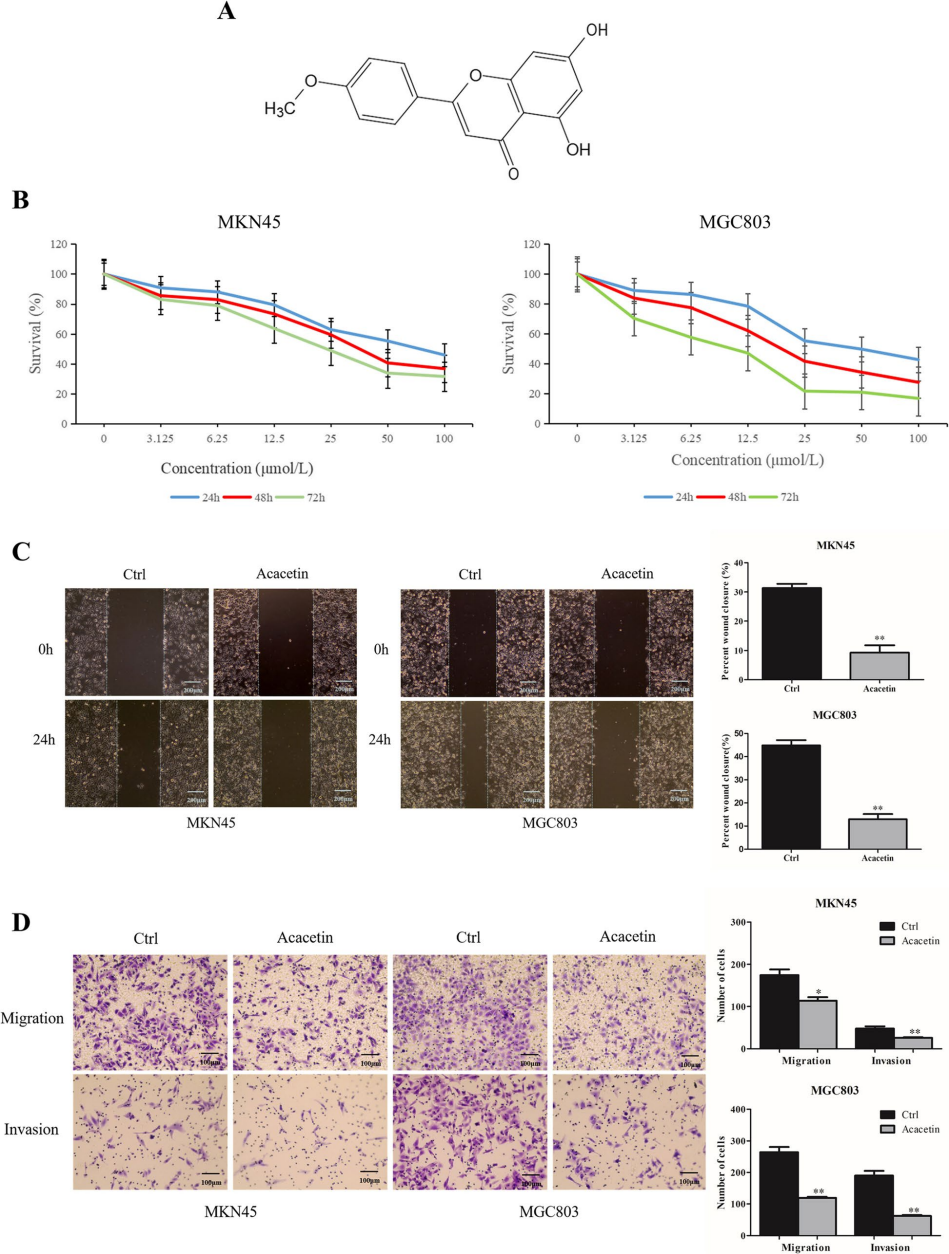
Effects of acacetin on the survival and invasion of MKN45 and MGC803 cells. **A** The chemical structure of acacetin. **B** MKN45 and MGC803 cells were treated with 0–100 μM acacetin for 24–72 h and subjected to CCK-8 assays. **C** A wound healing assay was performed to evaluate the antimetastatic effect of acacetin in MKN45 and MGC803 cells (magnification, 10×). **D** Transwell migration and Matrigel invasion assays of MKN45 and MGC803 cells treated with or without acacetin were performed (magnification, 20×)

We first investigated the effects of acacetin (Fig. 1A) on cell growth. GC cells were treated with different concentrations of acacetin from 0 to 100 μM for 24, 48 and 72 h, and cell viability was detected at different time points. As shown in Fig. 1B, the half maximal inhibitory concentrations (IC50) of MKN45 cells were 54.092 μM, 45.017 μM, and 36.961 μM at 24, 48 and 72 h, respectively. The IC50 of MGC803 cells was 48.357 μM, 33.449 μM, and 19.968 μM at 24, 48 and 72 h, respectively. Acacetin inhibited the proliferation of both MKN45 and MGC803 cells in a time- and dose-dependent manner. MGC803 cells were more sensitive to acacetin than MKN45 cells. Therefore, the optimal inhibitory concentration of MKN45 cells was 45 μM, and that of MGC803 cells was 33 μM.

The scratch assay is a well-developed method to detect cell migration *in vitro* [28]. In this study, we established two groups: a blank control group and an acacetin group. Acacetin (40 μM and 30 μM) was added to MKN45 and MGC803 cells, respectively. As shown in Fig. 1C (*p <* 0.05), the migration rate of GC cells was reduced after treatment with acacetin. In the cell invasion and migration assays, the number of cells migrating to the membrane in the acacetin group was significantly lower than that in the control group (*p <* 0.05, Fig. 1D). These results indicated that acacetin can inhibit the metastasis of GC cells.

### Acacetin regulated the expression of EMT-related biomarkers in MKN45 and MGC803 cells

**Fig. 2:**
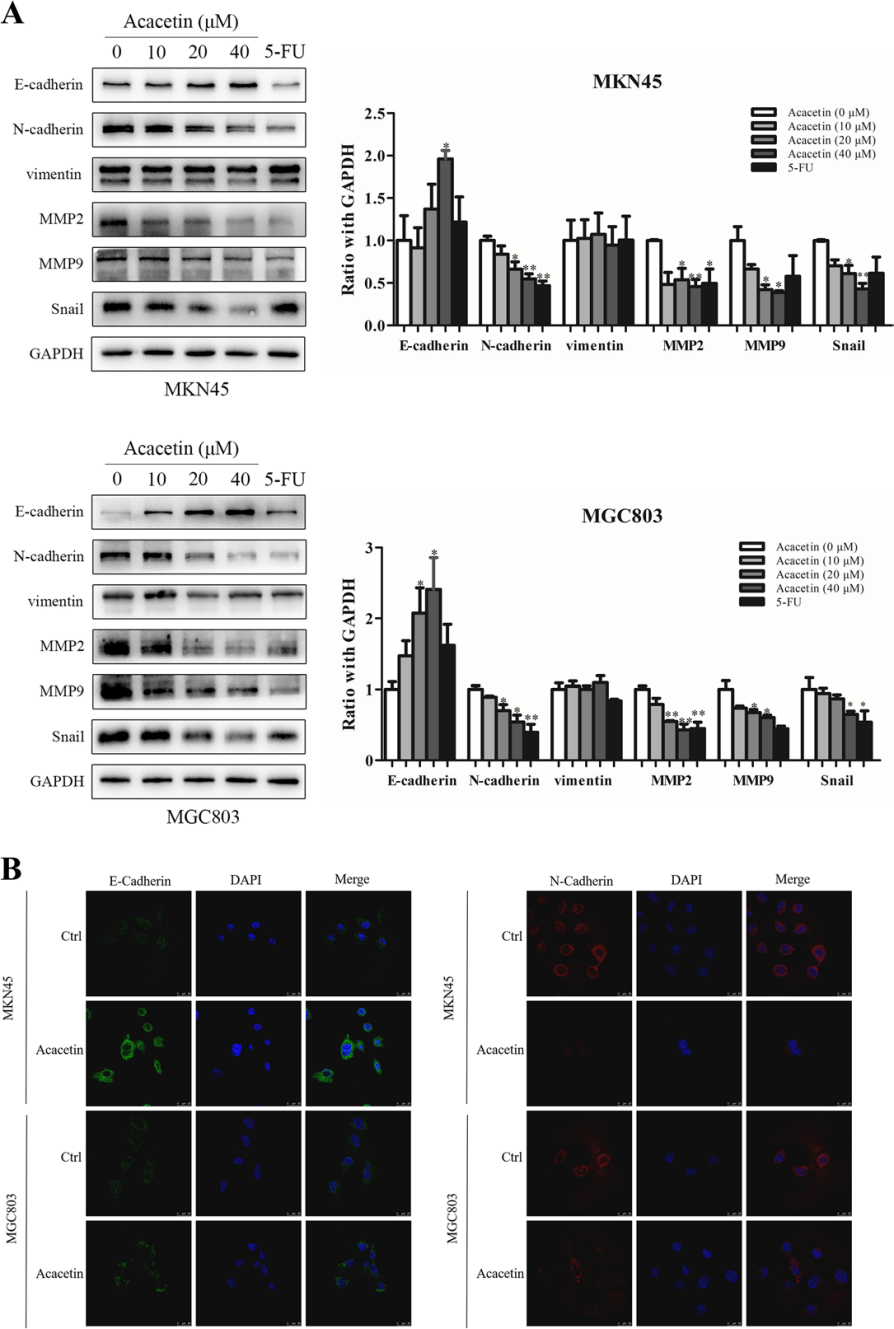
Effect of acacetin on EMT in MKN45 and MGC803 cells. **A** The protein expression levels of EMT biomarkers (E-cadherin, N-cadherin, Vimentin, MMP-9, MMP-2 and Snail) were determined by western blot analysis. **B** MKN45 and MGC803 cells were treated with acacetin for 24 h. Then, the cells were fixed and labelled for E-cadherin (green) and N-cadherin (red), and the nuclei were stained with DAPI (blue). The third parts show the merged images. Data are expressed as the mean ± SD (*n* = 3). * *p* < 0.05, ** *p* < 0.01 compared to the vehicle control

EMT is characterized by reduced E-cadherin expression and increased mesenchymal markers (such as N-cadherin and Vimentin), transcription factors (Twist, Slug and Snail) and MMPs [29]. In our study, GC cells were incubated with different concentrations of acacetin (10, 20, and 40 μm for MKN45 and 7.5, 15, and 30 μm for MGC803) for 48 h. The results showed that the expression of the epithelial marker E-cadherin was upregulated and that of the mesenchymal marker N-cadherin was downregulated, although Vimentin expression did not change (*p <* 0.05, Fig. 2A). In addition, compared with the control, acacetin treatment downregulated EMT-related Snail and metastasis-related MMP-2 and MMP-9 expression. Interestingly, some markers showed significant dose-dependent characteristics. In addition, immunofluorescence staining showed that E-cadherin expression was increased and N-cadherin expression was decreased in the GC cells treated with acacetin (*p <* 0.05, Fig. 2B). These findings indicated that acacetin inhibits EMT-related proteins.

### Acacetin inhibited TGF-β1-induced EMT of MKN45 and MGC803 cells

**Fig. 3:**
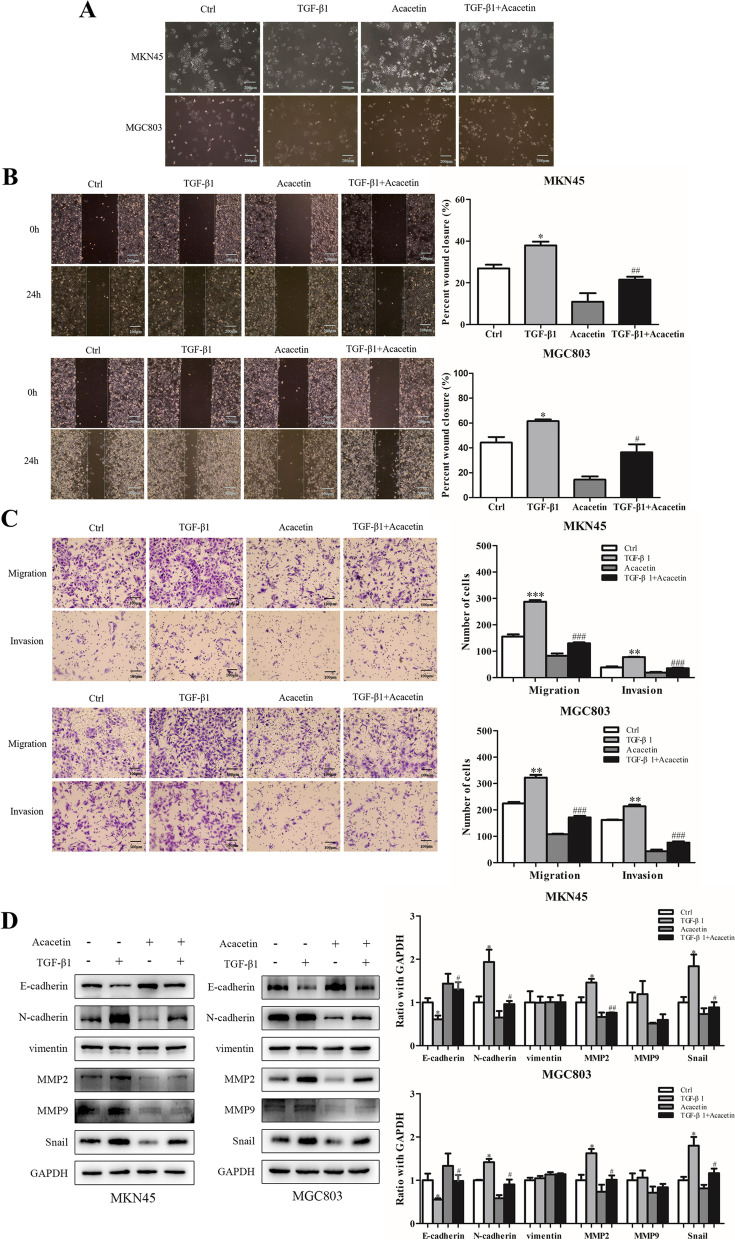
Effect of acacetin on invasion and migration in TGF-β1 (10 ng/ml)-treated MKN45 and MGC803 cells. **A** Morphological changes in MKN45 and MGC803 cells that were untreated or treated with acacetin. **B** Wound healing assays detected the effect of acacetin on TGF-β1 (10 ng/ml)-induced invasion of GC cells. **C** Transwell assays measured invasion and migration influenced by acacetin in GC cells under TGF-β1 treatment. **D** Western blot analysis of the protein changes in TGF-β1-induced EMT in GC cells. The quantitative values are expressed as the mean *±* SD (*n* = 3). * *p* < 0.05, ** *p* < 0.01, *** *p* < 0.001 compared to the control group. # *p* < 0.05, ## *p* < 0.01, ### *p* < 0.001 compared to the TGF-β1 alone group

TGF-β1 can activate EMT in a variety of tumour cells [30]. Therefore, we explored whether acacetin can influence TGF-β-induced EMT in GC cells. First, we investigated the influence of acacetin on the morphology of MKN45 and MGC803 cells. The results indicated that the morphological changes from epithelioid to mesenchymal in the TGF-β1 (10 ng/ml)-treated cells were reversed after the administration of acacetin, and monomer alone did not affect the appearance of the cells (Fig. 3A). Next, wound healing and Transwell migration assays were used to assess the effects of acacetin on TGF-β1 (10 ng/ml)-induced invasion and migration. Acacetin attenuated TGF-β1-activated migration of GC cells (*p <* 0.05, Fig. 3B). Cell migration to the chambers was inhibited (*p <* 0.05, Fig. 3C). Finally, in the TGF-β1 (10 ng/ml)-treated GC cells (*p <* 0.05, Fig. 3D), acacetin influenced protein expression, which was reflected in the suppression of mesenchymal biomarkers (N-cadherin, MMP-2 and Snail) and the enhancement of epithelial markers (E-cadherin). These results indicated that acacetin can reverse TGF-β1-activated EMT.

### Acacetin reversed activation of the Akt pathway in MKN45 and MGC803 cells

**Fig. 4:**
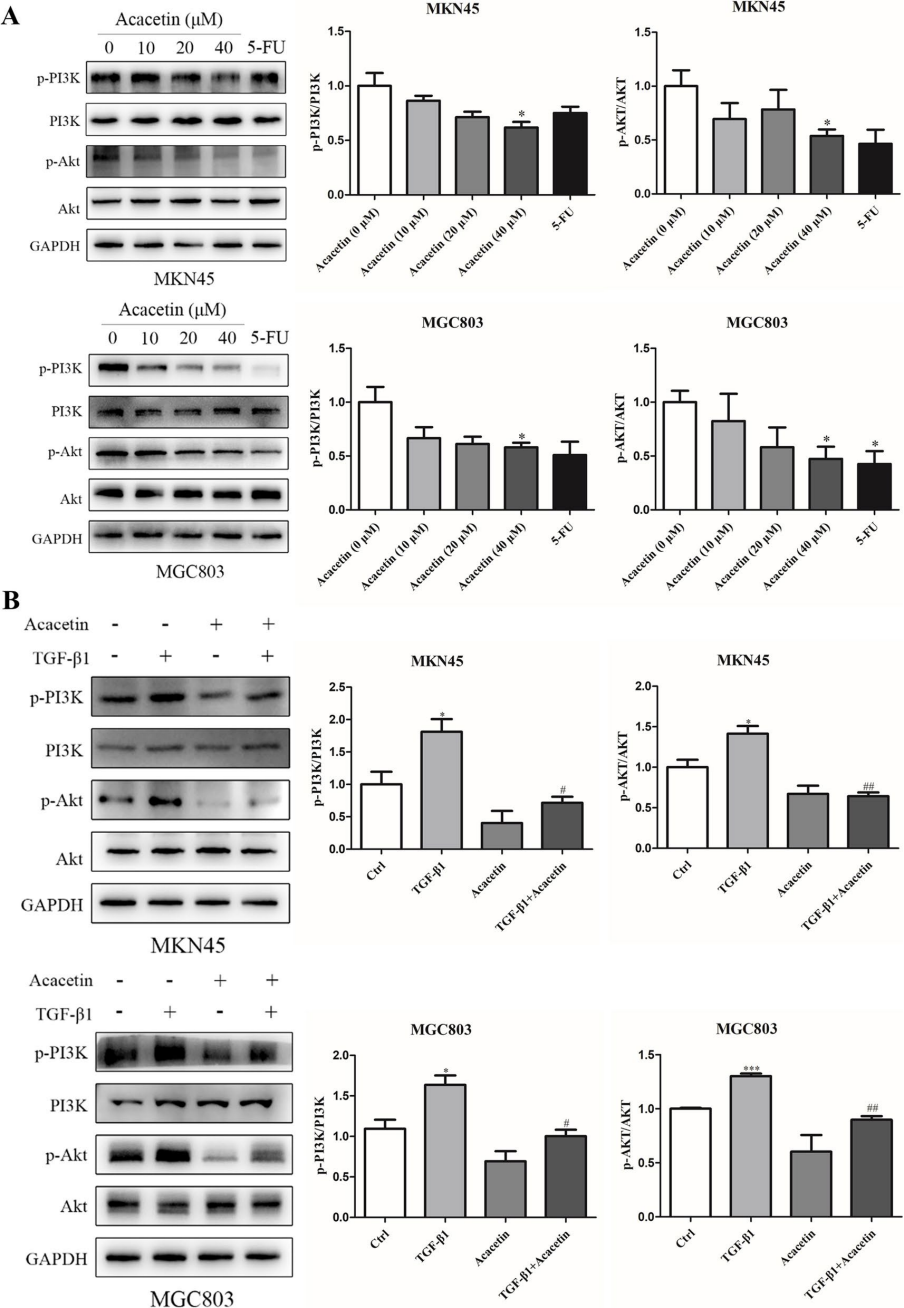
Effect of acacetin on the phosphorylation levels of PI3K and Akt in TGF-β1 (10 ng/ml)-treated GC cells. **A** Western blot analysis of the phosphorylation of PI3K and Akt in GC cells treated with acacetin. **B** GC cells were treated with TGF-β1 (10 ng/ml) in the absence or presence of acacetin. The quantitative values are expressed as the mean *±* SD of three independent experiments. * *p* < 0.05, ** *p* < 0.01, *** *p* < 0.001 compared to the control group. # *p* < 0.05, ## *p* < 0.01 compared to the TGF-β1 alone group

PI3K/Akt regulates cell proliferation and migration through a variety of mechanisms [12]. To explore the potential molecular mechanism by which acacetin inhibits invasion and migration in GC cells, we examined the effect of acacetin on the PI3K/Akt pathway. As shown by the western blot results in Fig. 4A (*p <* 0.05), acacetin reduced the phosphorylation of PI3K and Akt in a dose-dependent manner. Previous studies have shown that TGF-β can activate the Akt pathway [31, 32]. Then, we analysed the effects of acacetin on the TGF-β-activated Akt pathway. As presented in Fig. 4B (*p* < 0.05), acacetin decreased the expression levels of p-PI3K and p-Akt, which were increased by TGF-β1 (10 ng/ml). These findings suggested that the PI3K/Akt signalling pathway may be the mechanism by which acacetin inhibits the metastasis of GC cells.

### Effect of acacetin on peritoneal metastasis *in vivo*

**Fig. 5:**
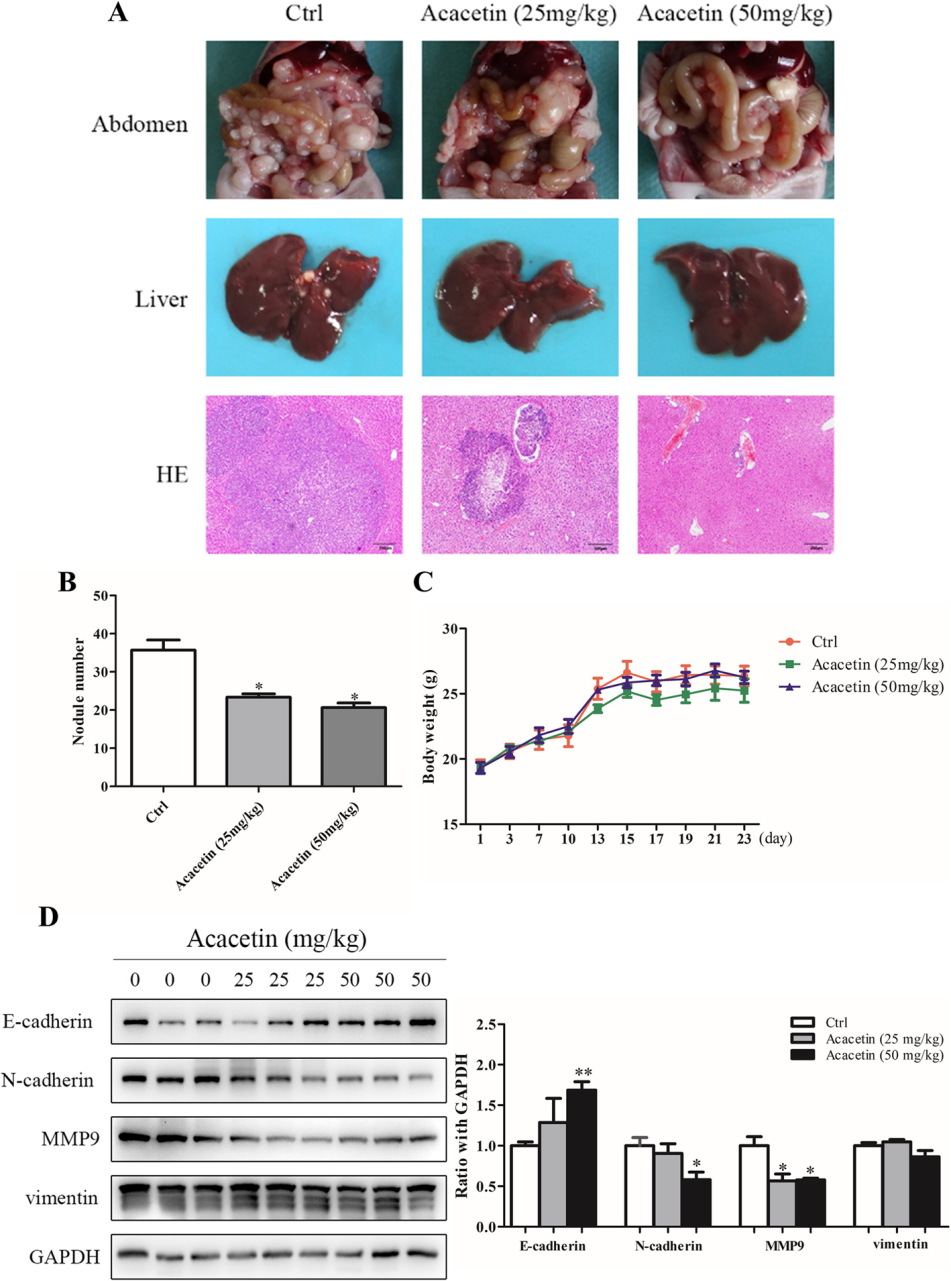
Mechanism of acacetin in peritoneal metastasis of GC in nude mice. **A** Tumours were implanted in the abdomen, and then, the gross and microscopic pathological features of metastatic nodules in the livers of mice were observed. **B** The number of cancerous nodules colonizing the abdomen. **C** Changes in body weight in nude mice. **D** Difference in EMT-related protein expression in liver metastatic nodules of mice treated with different concentrations of acacetin. Data are expressed as the mean *±* SD (*n* = 3). * *p* < 0.05, ** *p* < 0.01, *** *p* < 0.001 compared to the control group

To further determine the effects of acacetin in inhibiting GC metastasis, we established a peritoneal metastasis model in nude mice. The results showed more tumour nodules in the abdomen and metastatic tumour nodules in the livers of the mice in the acacetin low-dose and high-dose groups than those in the control groups (*p <* 0.05, Fig. 5A, B). Haematoxylin-eosin staining of the liver showed larger metastatic nodules in the control group, while only a small lump of neoplastic tissues adjacent to the blood vessels was found in the acacetin high-dose group (Fig. 5A). There was no significant difference in body weight among the groups (*p <* 0.05, Fig. 5C). Then, we detected the expression of EMT-related proteins using hepatic metastatic nodules. The western blot results indicated that the expression of N-cadherin and MMP-9 was decreased and that of E-cadherin was increased, whereas the expression of Vimentin in the acacetin groups did not change significantly compared with that in the control group (*p <* 0.05, Fig. 5D). These results indicated that acacetin can inhibit the metastasis of GC *in vivo*.

## Discussion

The objective of this study was to investigate the effect of acacetin on TGF-β1-induced EMT in GC cells and explore its possible mechanism. *In vitro* experiments showed that acacetin inhibited the invasion and migration of GC cells by regulating EMT-associated proteins. This effect was further confirmed in TGF-β1-induced EMT models. Subsequent experiments proved that acacetin inhibited the phosphorylation of PI3K and Akt in MKN45 and MGC803 cells and then activated the PI3K/Akt pathway. Acacetin also reversed the phosphorylation of PI3K and Akt. *In vivo* experiments showed that acacetin inhibited the growth of peritoneal tumours. Further analyses showed that acacetin regulates EMT-related proteins in hepatic metastatic nodules and suppresses liver metastasis.

The mortality of GC is higher and the prognosis is poorer due to recurrent disease, even in patients with surgical resection. GC easily metastasizes to other parts of the body, and the peritoneum is the most common site of metastasis [4]. Therefore, inhibiting tumour metastasis can improve the survival of GC patients. EMT is a crucial biological process that facilitates cancer metastasis [33]. The present study found that acacetin inhibits the invasion and migration of GC cells. The effect confirmed by subsequent trials was achieved by altering the expression of EMT-related proteins. The results showed that acacetin downregulated the expression of N-cadherin and upregulated the expression of E-cadherin. Members of the MMP family, MMP2 and MMP9, play a key role in tumour invasion, metastasis and angiogenesis [34]. We also found that acacetin reduced the expression of MMP2 and MMP9 in MKN45 and MGC803 cells. This result suggested that the inhibitory effects of acacetin on GC metastasis were achieved by controlling the expression of MMPs. Snail, a critical transcriptional repressor of E-cadherin, plays an important role in oncogenesis and EMT [35]. Our research found that acacetin significantly suppressed the expression of Snail in GC cells.

In the TGF-β1-induced EMT models of MKN45 and MGC803 cells, acacetin reversed the morphology of mesenchymal-like spindle-shaped cells and reduced invasion and migration stimulated by TGF-β1. Additionally, acacetin attenuated the overexpression of the mesenchymal marker N-cadherin, the transcription factor Snail and MMP2 and MMP9, although acacetin promoted the expression of the epithelial marker E-cadherin, which was reduced by TGF-β1. These results indicated that in TGF-β1-treated GC cells, acacetin inhibited EMT and repressed invasion and migration.

TGF-β can activate a variety of signalling pathways, such as Smad, ERK, p38 MAPKs, PI3K and RAS [36]. The PI3K/Akt pathway plays a key role in TGF-β-induced EMT of GC cells, and the phosphorylation of PI3K and Akt is increased during the progression of GC [37, 38]. In this study, we first examined the effects of acacetin on different signalling pathways, such as p38 MAPKs, through western blotting. We found that acacetin suppressed the phosphorylation of PI3K and Akt. Then, in the activated PI3K pathway, acacetin abrogated the upregulation of p-PI3K and p-Akt expression. Collectively, these results suggested that acacetin may inhibit TGF-β1-induced EMT of GC cells by inhibiting the PI3K/Akt signalling pathway. The detailed molecular mechanism requires further study.

As previously mentioned, peritoneal metastasis is the most common type in GC, occurring in approximately 10–30% of newly diagnosed patients, and more than 50% of patients in stage II–III developed peritoneal metastasis within 5 years after radical resection [39]. We select MKN45 cell line for animal experiment due to MKN45 originate from lymph node metastasis in a patient with gastric signet-ring cell carcinoma with poorly cohesive. In this study, we found that acacetin prohibits the growth of tumours in the abdomen. Furthermore, 4–14% of new cases with GC have liver metastasis [40], resulting in poor prognosis due to the lack of effective treatment. We also found that acacetin inhibits liver metastasis of GC cells. This effect is associated with the suppression of EMT. These results demonstrated that acacetin can control the development of peritoneal metastasis and inhibit liver metastasis of GC.

## Conclusions

In summary, our study showed that acacetin inhibits the migration, invasion and EMT of GC cells by suppressing the PI3K/Akt signalling pathway. Furthermore, we affirmed that acacetin inhibits the peritoneal metastasis and liver metastasis of GC. These results suggested that acacetin could be developed as a potential therapeutic agent for the treatment of metastatic disease of GC.

## Supplementary Information

**Additional file 1.**

**Additional file 2.**

**Additional file 3.**

**Additional file 4.**

**Additional file 5.**

**Additional file 6.**

**Additional file 7.**

**Additional file 8.**

## Data Availability

The data associated with this study are included in this published article. Additional files are available from the corresponding author upon reasonable request.

## Abbreviations

EMT: Epithelial-to-mesenchymal transition
TGF-β1: Transforming growth factor-β1
GC: Gastric cancer
CCK-8: Cell Counting Kit-8
MMP: Matrix metalloproteinase
DAPI: 4′,6-diamidino-2-phenylindole
IC50: Half maximal inhibitory concentration
