# Supplementary figures and images for: Acacetin inhibits invasion, migration and TGF-β1-induced EMT of gastric cancer cells through the PI3K/Akt/Snail pathway
Source: BMC Complement Med Ther. 2022 Jan 9;22:10. doi: 10.1186/s12906-021-03494-w (PMC8744305; doi:10.1186/s12906-021-03494-w)

Supplementary Figure 2: consistent with Figure 2 in the text.

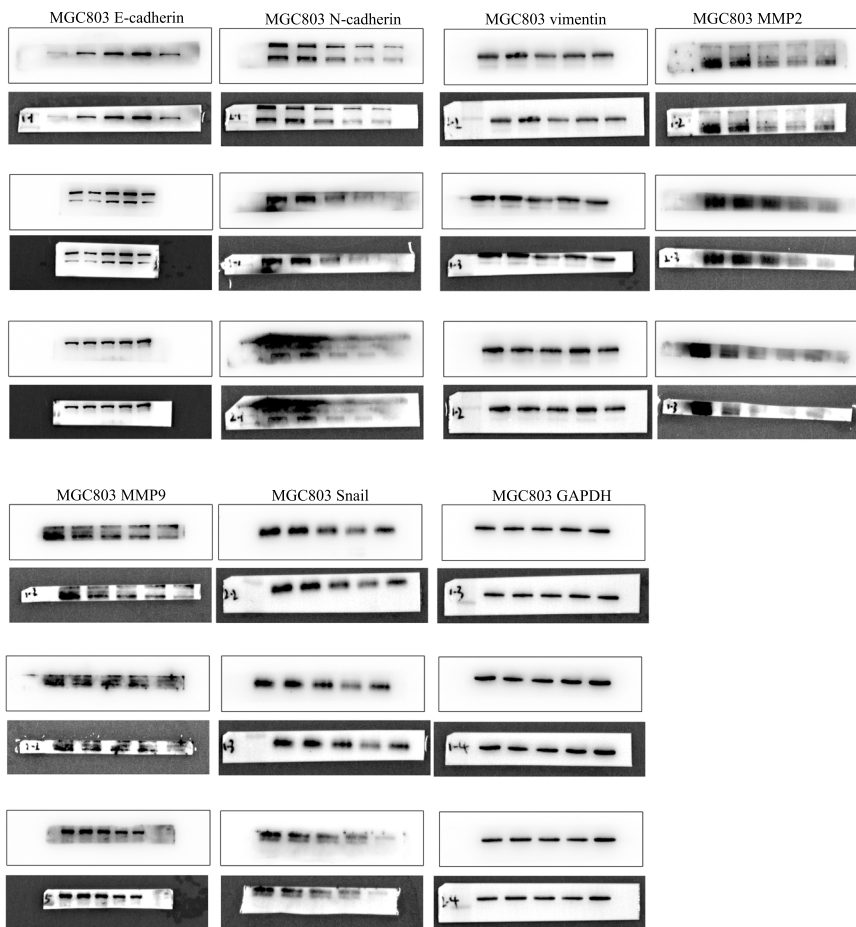

Supplement: Supplementary file 3 — Additional file 3. [file 12906_2021_3494_MOESM3_ESM.pdf]

Supplementary Figure 3: consistent with Figure 3 in the text.

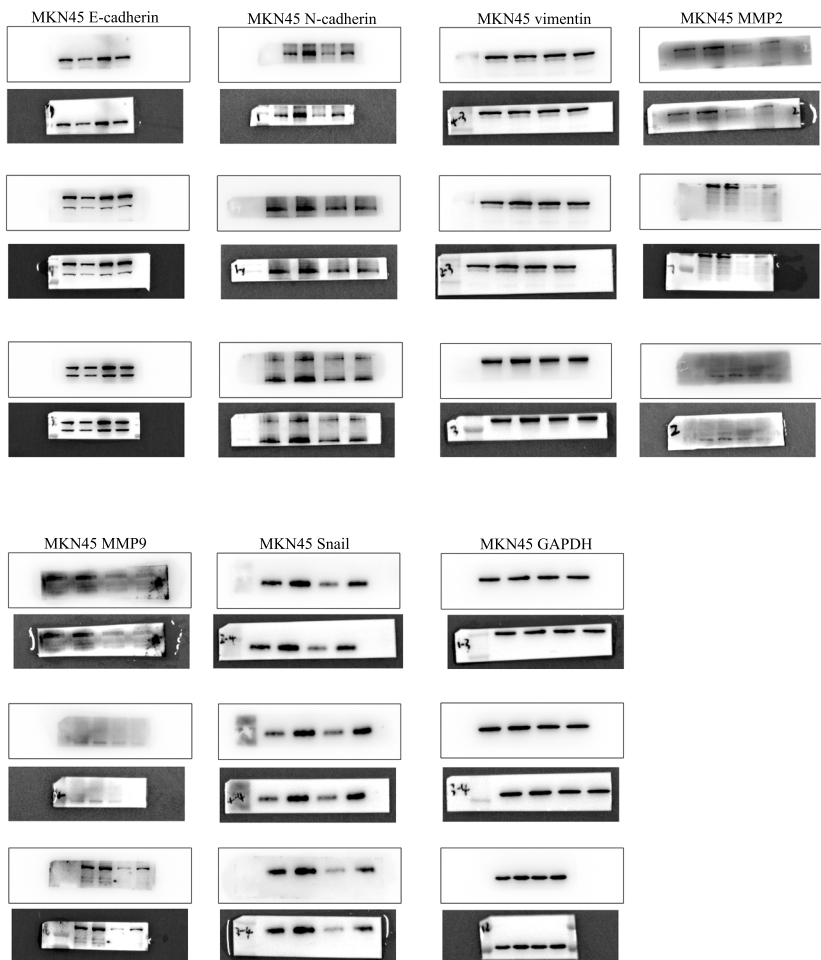

Supplement: Supplementary file 4 — Additional file 4. [file 12906_2021_3494_MOESM4_ESM.pdf]

Supplementary Figure 4: consistent with Figure 3 in the text.

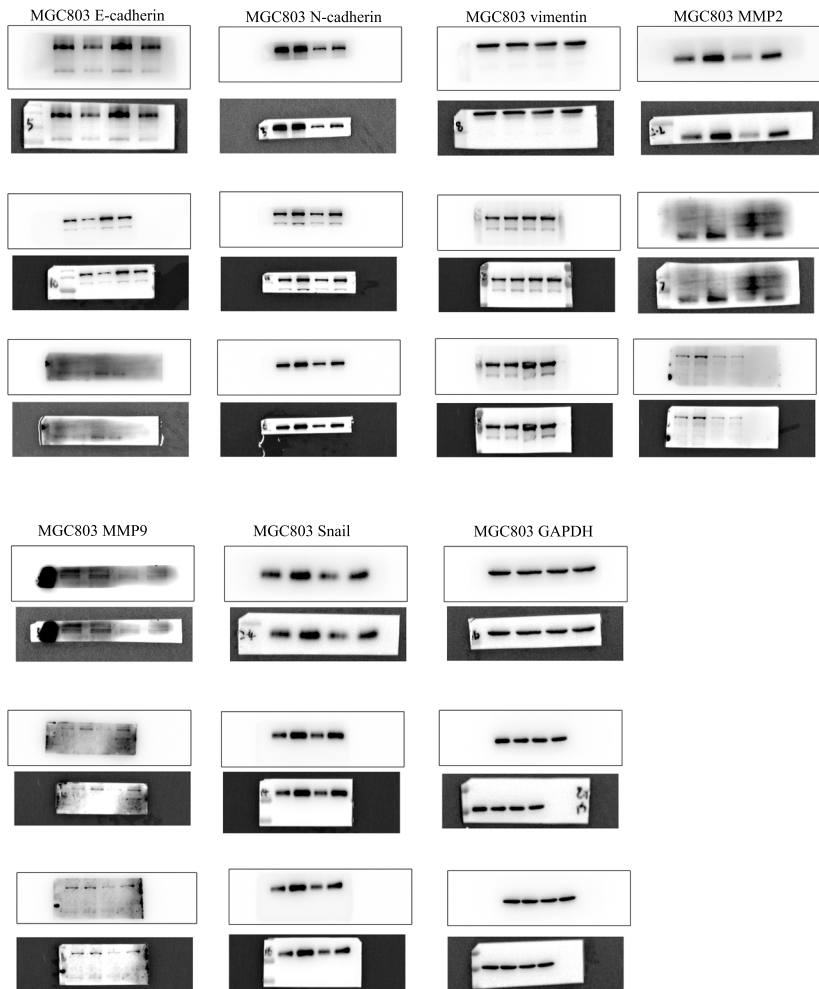

Supplement: Supplementary file 5 — Additional file 5. [file 12906_2021_3494_MOESM5_ESM.pdf]

Supplementary Figure 5: consistent with Figure 4A in the text.

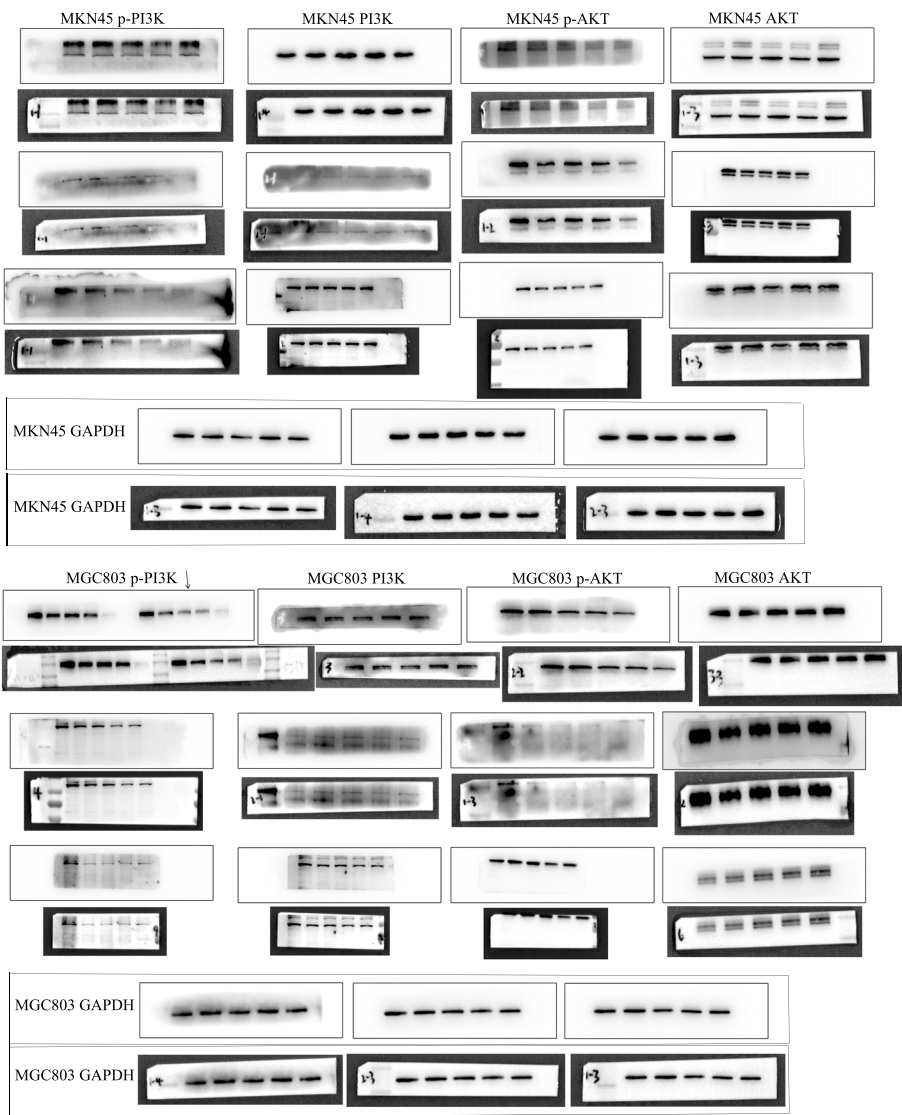

Supplement: Supplementary file 6 — Additional file 6. [file 12906_2021_3494_MOESM6_ESM.pdf]

Supplementary Figure 6: consistent with Figure 4B in the text.

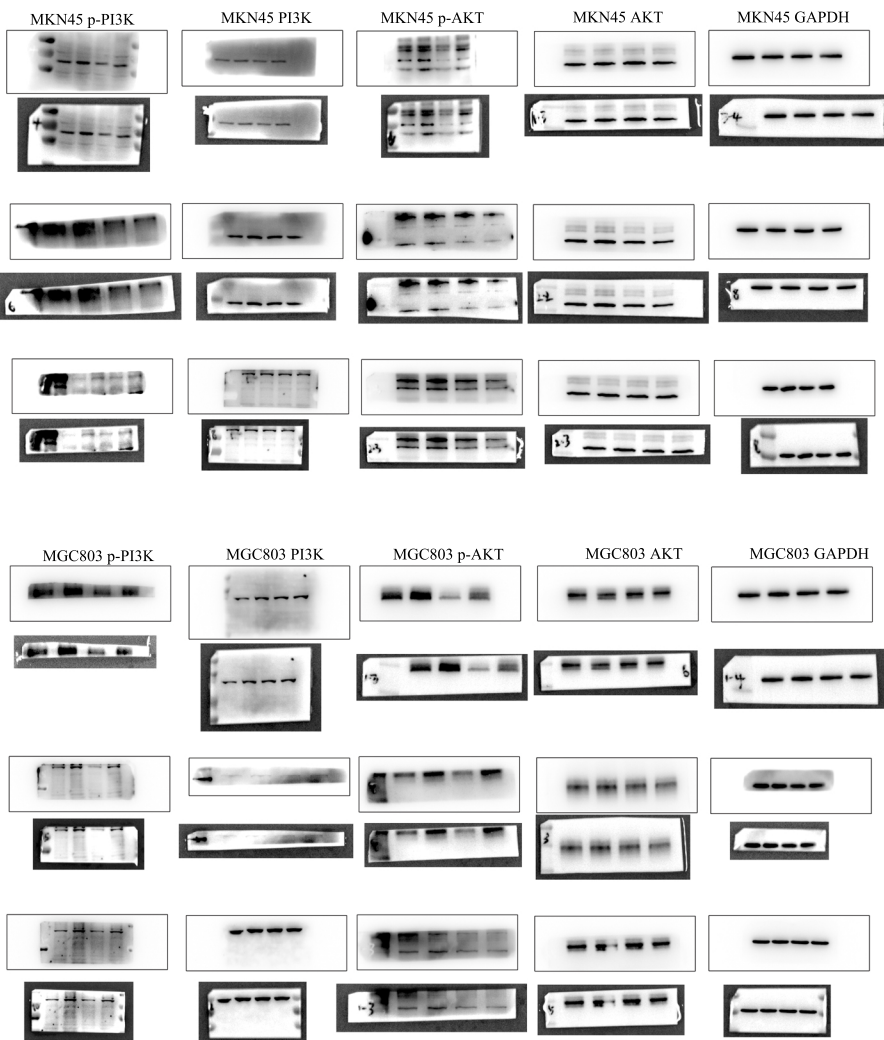

Supplement: Supplementary file 7 — Additional file 7. [file 12906_2021_3494_MOESM7_ESM.pdf]
