# Supplementary material for: Acacetin inhibits invasion, migration and TGF-β1-induced EMT of gastric cancer cells through the PI3K/Akt/Snail pathway
Source: BMC Complement Med Ther. 2022 Jan 9;22:10. doi: 10.1186/s12906-021-03494-w (PMC8744305; doi:10.1186/s12906-021-03494-w)

Supplementary Figure 7: consistent with Figure 5 in the text.

E-cadherin

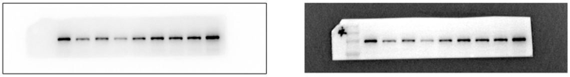

N-cadherin

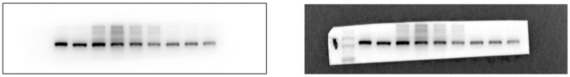

MMP9

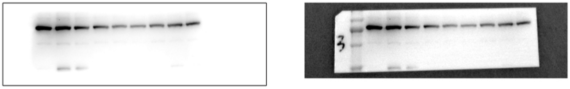

Vimentin

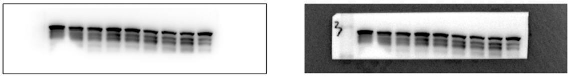

GAPDH

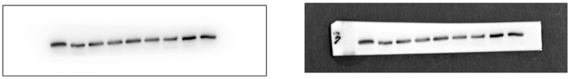

Supplement: Supplementary file 8 — Additional file 8. [file 12906_2021_3494_MOESM8_ESM.pdf]
